# Case report: Uridine triacetate in the management of delayed onset 5-fluorouracil toxicity: A case report and review of literature

**DOI:** 10.3389/fphar.2022.977734

**Published:** 2022-09-07

**Authors:** Aasems Jacob, Janeesh Sekkath Veedu, Insija Selene, Rishi Raj, Lakshmi Kannan, Reema Patel

**Affiliations:** ^1^ Department of Hematology and Oncology, Pikeville Medical Center, Pikeville, KY, United States; ^2^ Division of Medical Oncology, Department of Internal Medicine, University of Kentucky, Lexington, KY, United States; ^3^ Department of Endocrinology, Pikeville Medical Center, Pikeville, KY, United States; ^4^ Department of Nephrology, Pikeville Medical Center, Pikeville, KY, United States

**Keywords:** 5-fluorouracil, capecitabine, chemotherapy, toxicity, uridine triacetate, dihydropyridine deaminase deficiency, vistogard

## Abstract

5-fluorouracil (5FU) and capecitabine are fluoropyrimidine anti-neoplastic drugs commonly used in the treatment of different types of cancer. Hereditary dihydropyrimdine deaminase (DPD), thymidylate synthase mutations and drug overdose may lead to life-threatening toxicities. Uridine triacetate (UTA) is an emergency treatment for overdoses and early onset, severe or life-threatening toxicities from fluoropyrimidines. It is approved for use in adults and children within 96 h of last fluoropyrimidine administration. We present the case of a 64-year-old male treated with 5-FU and oxaliplatin as adjuvant systemic therapy for stage IIIA rectal cancer who developed delayed central nervous system toxicity 18 days after initiating chemotherapy. He had rapidly worsening encephalopathy and ataxia. Laboratory workups, MRI brain and EEG were negative. He was started on UTA with concerns of 5-FU toxicity due to the life-threatening nature of his condition even beyond the recommended 96-h time cut-off. He had rapid improvement in clinical status and resolution of encephalopathy. DPD deficiency testing later resulted as heterozygous for IVS14+1G>A allele indicating enzyme deficiency. This report demonstrates the importance of identifying delayed side effects with fluoropyrimidine therapy and potential treatment for reversing these effects. We also did an extensive literature review and obtained reports from the uridine triacetate clinical trials on patients receiving UTA after the 96-h cut-off. Based on our experience and previous published reports, a patient developing life-threatening delayed 5-FU toxicity should also be considered for UTA on a case-by-case basis.

## Introduction

5-Fluorouracil (5FU) or capecitabine, a prodrug enzymatically converted to 5-FU, is prescribed to thousands of patients with gastrointestinal, breast, and other cancers in the United States (US) (Ison et al., 2016). The National Institutes of Health (NIH) estimates that roughly 275,000 patients receive 5-FU every year, and 60,000 treatment packages of capecitabine are sold per year (Santos et al., 2017). Most patients experience adverse effects from these drugs, including fatigue, oral mucositis, abdominal pain, diarrhea, myelosuppression, palmoplantar erythrodysesthesia and rarely central nervous system toxicity or cardiomyopathy. However, serious toxicities are rare at a 3% incidence, causing around 1,300 deaths every year (Santos et al., 2017).

Dihydropyridine deaminase (DYPD) deficiency due to genetic variations predispose patients to the toxic effects of 5-FU and capecitabine by reducing deactivation which increases blood levels of the drug. High-risk polymoprhisms in the thymidylate synthase gene (TYMS) is also associated with higher risk of toxicity, although the exact mechanism is not certain. Early toxicity or significant side effects should raise suspicion for these genetic variations, necessitating prompt treatment. At least 3% of the general population and 3%–5% of White patients are partially deficient for DPYD enzyme activity while 0.2% have complete *DPYD* deficiency (He and Zhou, 2014). Pharmacogenomic testing helps in identifying patients who are at greater risk of toxicity. However, given that the prevalence of DPYD gene mutation is low, it is not cost-effective or time sensitive to test all patients receiving 5-FU based chemotherapy.

The use of pyrimidine nucleosides as antidotes in the treatment of adverse effects of fluoropyrimidines have been explored, but it is restricted to topical use for dermatologic side effects (Hartinger et al., 2012). In 2015, the United States Food and Drug Administration (FDA) approved uridine triacetate (UTA) as an emergency treatment for overdoses and early-onset, severe, or life-threatening toxicities from 5-FU (Wellstat Therapeutics Corporation, 2015). It is approved for use in adults and children within 96 h of fluoropyrimidine administration. The efficacy of this drug was shown in a pooled analysis of two clinical studies among 135 patients with fluoropyrimidine toxicity who received uridine triacetate within 96 h of administration of 5-FU or capecitabine (Santos et al., 2017). The use of the drug resulted in approximately 96% survival (compared to 16% survival in a historic cohort), and about half the patients resumed their chemotherapy regimens within 3 weeks of overexposure. Although acute onset toxicity and overdose are more common, delayed 5-FU toxicity is extensively reported and more often seen in clinical practice. We present the case of a patient with delayed life-threatening 5-FU toxicity who was successfully treated with UTA with administration greater than 96-h after the last 5-FU dose. Our case emphasizes the potential life-saving benefit of this antidote beyond its currently stipulated indications and to our knowledge, this is the first report of benefit of UTA among patients with delayed 5-FU toxicity.

## Case description

A 64-year-old male with a history of coronary artery disease was found to have a 2.5 cm rectal lesion in the posterior lateral wall on colonoscopy done to evaluate rectal bleeding. Biopsy showed intramucosal adenocarcinoma arising in high-grade dysplasia. Computerized tomography (CT) of the chest, abdomen, and pelvis showed mild rectosigmoid wall thickening. No lymphadenopathy or distant metastases were identified. He underwent abdominoperineal resection with pathology showing grade 2 invasive adenocarcinoma, 1.9 cm in size and located below anterior peritoneal reflection invading submucosa. Margins were uninvolved by the tumor, and 1 of the 65 lymph nodes removed showed involvement by tumor. The disease was staged as pT1N1M0- stage IIIA. He was initiated on adjuvant modified FOLFOX (5Fluorouracil 400mg/m^2^ IV bolus, leucovorin 400mg/m^2^, oxaliplatin 85mg/m^2^, continuous 5-Fluoruacil infusion 2400mg/m^2^ over 46 h every 14 days) with subsequent chemoradiation plans. He received the first cycle of chemotherapy and had only mild fatigue as a side effect. However, after the second cycle, he started developing transient episodes of gait instability and memory loss. The symptoms worsened over the next 2 weeks, and he sought medical attention after a fall. Evaluation in the emergency department revealed lack of coordination in bilateral upper and lower extremities, horizontal nystagmus on lateral gaze, and confusion. His neurological symptoms worsened over the next 24 h, progressive lethargy requiring endotracheal intubation for airway protection. He was 16 days from the last 5FU. Thiamine was given empirically. CT angiogram of head and neck, and Magnetic Resonance Imaging (MRI) of brain did not show acute intracranial abnormalities. Electroencephalogram showed continuous bi-hemispheric slowing suggestive of moderate encephalopathy without epileptiform activity. Cerebrospinal fluid analysis, ammonia levels, toxicology analysis of urine and blood as well as infectious disease workup were normal. Despite thorough workup and supportive management, there was no improvement in mental status as sedation was weaned weaning sedation. By exclusion he was assumed to have possible delayed 5-FU toxicity. Although the current FDA approval is for use within 96 h of last treatment, as a last resort, the patient was administered uridine triacetate 10 g every 6 h for a total of 20 doses through orogastric tube. His neurological status improved within two doses of receiving the antidote and was extubated after 24 h. On completion of the full 20 doses, he had complete neurological recovery with resolution of gait instability and coordination defects. DPD deficiency testing was sent on admission and was heterozygous for IVS14+1G>A allele. His adjuvant treatment was completed with radiation alone as per patient preference. Follow up scans after 3 months of treatment showed no evidence of disease. His neurological or physical strength is back to baseline after undergoing subacute rehabilitation and physical therapy.

## Discussion

Central Nervous System (CNS) toxicity from fluoropyrimidines is rare and symptoms can include somnolence, altered consciousness, and cerebellar ataxia, occurring in about 5% of patients (Shehata et al., 1999). Other adverse effects like peripheral neuropathy, cerebellar dysfunction (Choi et al., 2001), cerebral demyelination (Fassas et al., 1994), and leuko-encephalopathy (Hook et al., 1992) have been reported. Animal studies and autopsy findings suggest this could be due to delayed myelin destruction in the CNS (Choi et al., 2001; Han et al., 2008). 5-FU readily crosses blood-brain barrier with highest concentrations found in the cerebellum. Hence, the commonly reported neurotoxicity is a slowly progressing cerebellar syndrome with ataxia, dysmetria, dysarthria and nystagmus, the onset of which can be delayed for weeks to months after initiation of treatment (Takimoto et al., 1996; Shehata et al., 1999; Choi et al., 2001; Mitani et al., 2017). Encephalopathy often follows acute cerebellar syndrome or posterior reversible encephalopathy syndrome (PRES) (Dedić Plavetić et al., 2014). Findings of hyperammonemia on labs and diffuse periventricular white matter changes in the T2-weighted magnetic resonance imaging may be seen (Choi et al., 2001; Di Federico et al., 2020). However, these findings are not always present and were not evident in our patient. Development of encephalopathy is associated with poor prognosis and has near 100% fatality (median survival: 4.3 months) due to irreversible cerebellar damage (Suresh et al., 2015; Mitani et al., 2017). CNS related adverse events differ from gastrointestinal or hematologic side effects which often resolve with discontinuation of the drug.

The antidote drug, UTA is deacetylated into uridine in the body and work by competing with fluorouridine triphosphate, a 5-FU metabolite for incorporation into RNA of cells to reduce toxicity in normal tissues (Hidalgo et al., 2000). The benefit of the drug was proven in 2 open-label clinical studies among patients with acute early-onset toxicity and those with fluoropyrimidine overdose (Ison et al., 2016; Ma et al., 2017). This inclusion criteria were reportedly selected after review of historic case reports and FDA Adverse Event Reporting data (Ison et al., 2016). Ten out of the 177 patients developed life-threatening encephalopathy or mental status changes and 8 survived with UTA administration. Most common side effect with UTA include nausea (4.6%), vomiting (8.1%) and diarrhea (3.5%) and were mild to moderate. When both studies were analyzed, out of all the 10 deaths, 5 were in patients receiving UTA after 96 h (including 2 patients with overdose), while 5 deaths were attributed to cancer progression, tumor lysis or septic shock. The cut-off of use within 96-h of last fluoropyrimidine dose was recommended based on this report and preclinical studies (Saif and von Borstel, 2006; Borstel et al., 2010). However, due to this specific criterion used in the study, many patients who may have derived benefit from the drug including patients with delayed 5-FU toxicity often reported among those with DYPD deficiency and overdose may be precluded.

With the mechanism of toxicity similar in both acute and delayed onset toxicity, it is expected that UTA may be effective in both scenarios. Our case along with the previous reports by Zurayk et al. (2019), Baldeo et al. (2018) and Leung et al. (2021) signifies the importance of treatment of 5-FU toxicity with UTA even after the predefined 96 h. We obtained clinical characteristics and details of outcomes of patients (*n* = 13) who received UTA after the predefined 96-h window from the sponsors of the clinical trial and by review of literature (Table 1). All the cases, except ours, reported acute toxicity within a week of first cycle of fluoropyrimidine administration. Nine patients had gastrointestinal side-effects, 11 had hematologic toxicity, 8 developed encephalopathy and 2 patients had cardiac side effects. Eight patients survived while 5 died from complications of toxicity. Although this points to the need of early diagnosis and initiation of UTA, it is very clear that many patients treated beyond the 96-h window of last chemotherapy based on clinical judgement derived benefit, often recovering from life-threatening complications.

**TABLE 1:** Cases reporting use of uridine triacetate after the predefined 96-h window after last 5-FU or capecitabine dose.

Study/Data obtained from	Age	Fluoropyrimidine dose	Presenting symptoms (onset)	Hours after chemo UTA was used, doses	Genetic testing	Outcome
This report	64	5160 mg 5-FU over 46 h x 2 cycles	Gait instability, memory loss, lethargy, encephalopathy (18 days after starting chemotherapy)	384 h, 20 doses	Heterozygous c.1905+1G>A variant	Survived (response in 12 h)
Leung, et al. (Leung et al., 2021)	52	2400 mg/m^2^ 5-FU over 46 h	N/V/D, mucositis, fatigue, neutropenia, anemia (6 days after first cycle)	480 h, 20 doses	DPYD p.Y186C	Survived
Ma, et al. (Ma et al., 2017), WT	49	5542 mg 5-FU over 71 h	N/V, diarrhea, mucositis, encephalopathy, cardiogenic shock, thrombocytopenia	107.1 h, 20 doses	Not done	Survived
Ma, et al. (Ma et al., 2017), WT	59	5320 mg 5-FU over 46 h	Septic shock, AKI, encephalopathy, pancytopenia, multiorgan failure	101.2 h, 20 doses	Homozygous DPYD, TYMS mutations present	Died (Day 19)
Ma, et al. (Ma et al., 2017), WT	49	7250 mg 5-FU over 94.75 h	N/V, diarrhea, mucositis, abdominal pain, neutropenia	137 h, 4 doses	Negative for IVS14+1G-A	Died
Ma, et al. (Ma et al., 2017), WT	42	5140 mg 5-FU over 46 h	N/V, diarrhea, mucositis, encephalopathy, pancytopenia, alopecia	>520 h, 20 doses	DPYD, TYMS mutations present	Survived
Ma, et al. (Ma et al., 2017), WT	66	6800 mg 5-FU over 96 h	N/V, diarrhea, mucositis, encephalopathy, pancytopenia	282.5 h c, 8 doses	DPYD, TYMS mutation present	Died
Baldeo, et al. (Baldeo et al., 2018)	56	6320 mg 5FU over 96 h	Diarrhea, abdominal pain, mucositis within 24 h, pancytopenia	168 h, 20 doses	TYMS 2R/3RC genotype	Response in 12 h, Survived
Stokkeland, et al. (NACCT, (2019))	68	4000 mg/m^2^ 5FU over 96 h	Bloody diarrhea 3 days after last infusion. Mucositis, renal failure, cardiac ischemia, infection, pancytopenia (3 days after first cycle)	120 h, 20 doses	TYMS 2R/3RC genotype	Survived
Ma, et al. (Ma et al., 2017), WT	41	12,000 mg capecitabine over 4 days	N/V, mucositis, encephalopathy, neutropenia	97.5 h, 14 doses	Not done	Died (Day 11)
Ma, et al. (Ma et al., 2017), WT	63	26,000 mg capecitabine over 6 days	Mucositis, epidermal necrolysis	168 h, 20 doses	None detected	Survived
Ma, et al. (Ma et al., 2017), WT	78	18,000 mg capecitabine over 5 days	Mucositis, erythema, encephalopathy, pancytopenia	168 h, 9 doses	DPYD, TYMS mutations present	Died (Day 12)
Zurayk, et al. (Zurayk et al., 2019)	57	6800 mg/m2 capecitabine daily over 4 days	N/V, diarrhea, mucositis, diarrhea, n/v, fever, encephalopathy, pancytopenia (onset after 4 days)	504 h, 20 doses	Homozygous for c.1905+1G>A (*2A) variant	Survived (response in 6 days)

Abbreviations: WT, Wellcare Therapeutics; N/V/D, Nausea/vomiting/diarrhea, AKI, acute kidney injury; DPYD, dihydropyrimidine deficiency; TYMS- thymidylate synthase.

In our case, we made a clinical decision to test this patient for DPYD deficiency and start UTA due to the near 100% likelihood of fatality without intervention. UTA replenishes intracellular uridine that counteracts the effects of 5-FU, regardless of any genetic mutation (Zurayk et al., 2019). Based on our experience, a patient developing severe delayed 5-FU toxicity should be considered for UTA on a case-by-case basis. This is especially important for those with cardiac or neurologic toxicities which are often irreversible and life-threatening. Per FDA guidelines, all patients with fluoropyrimidine overdose received UTA within 96 h of last dose, even before onset of symptoms. In clinical practice it is possible that the dosing error or accidental/suicidal ingestion goes undetected, and patients may start developing toxicity symptoms days to weeks later. We believe these patients who develop severe symptoms might also benefit from UTA despite being out of the 96-h window. The cost of the drug (approximately USD 4815.60 per 10 g dose) and FDA labeling are limitations for the use of the drug in many of these unique and life-threatening scenarios. In acute toxicity, the 96-h cut-off may still be relevant as the antidote may not be effective in reversing clinical status once severe sepsis, multi-organ failure or irreversible neuronal damage occurs. We also acknowledge the possibility of underreporting of cases which did not derive benefit from UTA administration after 96-h window and hence multicenter clinical trials and observational studies are needed to assess clinical benefit.

In our opinion, it is important for oncologists to identify the life-threatening side effect from fluoropyrimidines which can present early or delayed and often result in death if untreated. Physicians should consider using UTA in patients developing life-threatening complications from the toxicity early in the course, and delayed use may be considered on a case-by-case basis. It should also be a priority to perform larger studies to determine efficacy of uridine triacetate among patients with delayed 5-FU toxicity.

## Data Availability

The original contributions presented in the study are included in the article/Supplementary Material; further inquiries can be directed to the corresponding author.
